# Cessation of Care for Frail Older Adults: Physical, Psychological and Economic Outcomes for Family Carers

**DOI:** 10.3390/ijerph19063570

**Published:** 2022-03-17

**Authors:** Aviad Tur-Sinai, Dafna Halperin, Nissim Ben David, Ariela Lowenstein, Ruth Katz

**Affiliations:** 1 Department of Health Systems Management, The Max Stern Yezreel Valley College, Yezreel Valley 1930600, Israel; dafnah@yvc.ac.il; 2 School of Nursing, University of Rochester Medical Center, Rochester, NY 14627-0446, USA; 3 Department of Economics, The Western Galilee Academic College, Akko 2412101, Israel; nissim.bendavid@gmail.com; 4 Department of Gerontology, Head Social Gerontology, Center for Research and Study of Aging, University of Haifa, Haifa 3498838, Israel; ariela@research.haifa.ac.il; 5 Department of Human Services, The Max Stern Yezreel Valley College, Yezreel Valley 1930600, Israel; ruth@soc.haifa.ac.il

**Keywords:** informal care, cessation of care, family care outcomes, SHARE

## Abstract

Global population aging and increased longevity are making family care a nearly universal experience. Caregiving is a dynamic process that varies over time and in intensity but often takes a physical and emotional toll on carers and may inflict financial costs by attenuating their labor market participation. The study explores the implications of the ‘cessation of care’ of frail elders by adult (middle-aged and older) kin by comparing two ethnic groups in Israel with respect to their health and their psychological and economic life. Using secondary data analyses based on SHARE-Israel data for persons aged 50+, it is found that subjective health assessment and financial capability are significantly higher among those who stop providing care than among those who continue to do so, while carers report a downturn in life satisfaction after they stop giving care. Those who continue are younger than the others, and their labor force participation rate is higher. Significant implications of cessation of care for all three areas studied—psychological, health, and economic—are found as well: the subjective rating of health and financial capability improve whereas life satisfaction decreases. Furthermore, a cessation of care moderates the relation between individuals’ age and their self-rated health, which is better among those who continue to provide care. These results emphasize and deepen our understanding of the cessation-of-care phase as a key component of the process of care for frail older adults by family members.

## 1. Introduction

The overarching goal of this study is to explore cessation-of-care outcomes in terms of the health, psychological, and economic wellbeing of female and male carers. The conceptual framework used is the ‘intergenerational solidarity, conflict, and ambivalence’ model [1].

Given global population aging and increased longevity, with the oldest of the old—the 85+ cohort—as the fastest-growing segment, family care is becoming a nearly universal experience [2]. Caregiving is a dynamic process that varies over time and in intensity [3]; it often takes a physical and emotional toll on carers [4] and may inflict financial costs on them by crimping their labor market participation [5]. Thus, contemporary families enter into care relationships that are new in terms of intensity and duration, necessitating renegotiation of intergenerational relations [6]. Mid-lifers are focal in this inquiry because they stand at the crossroads of generational and gender priorities and must reconcile obligations within and between care and other areas of life [7]. 

Frail elders are the most significant consumers of health resources in both acute and community services. Formal systems and families are both involved in such care. With the increase in life expectancy and the slow retreat of state responsibility, the carer is becoming a life-course role identity, one that a person is likely to enter and exit once or more in adulthood [8,9,10]. 

In contrast to the abundance of research on the impact of caring on carers (e.g., [9,11,12]), data on cessation of care and its aftermath remain quite sparse and limited. The current study aims to fill some of this gap. We define *cessation of care* as the period when middle age and older adults who were primary carers for older family members terminate their care relations. Long-term elder care ends in one of four ways: with the death of the care recipient [13], with his or her move to an institutional setting [14], when another family carer takes over [15], or as the need for care diminishes [16]. 

The period commencing with cessation of care requires further attention, particularly because some data point to the difficulty in adjustment and lack of support for carers when caregiving ends [17,18,19]. To fully understand cessation-of-care outcomes, one must consider the following: developmental timing of transitions; parallel trajectories in other realms of the carer’s life (health, family, career); and factors that moderate or mediate adjustment once care has ended [20]. To investigate these, secondary quantitative analyses of data from two waves (survey rounds) of the SHARE-Israel study were used to build two care configurations: continuing or ceasing. The use of these data may be instructive of changes in carers’ characteristics over time and reveal the existence or nonexistence of significant effects on the carers’ physical, psychological, and economic outcomes due to the change in their caring position.

Caregiving lies at the intersection of three institutions that interact to shape care experiences: family, workplace, and the government sector. Although most primary carers are still women, changes in family composition and women’s increased participation in the labor force are reducing the supply of potential carers [2,21,22]. The prevalence of caregiving among adult women is high when examined as a lifetime risk. These trends are bound to augment the burden on families and states—two major pillars of support in old age. As families will have to rely on a decreased pool of informal carers [23], demand for formal care and more workplace flexibility is likely to grow. With state spending under increasing constraints, long-term elder care has become a major issue for families and states. Government incentives and policies, along with workplace policies and changing family structures, are producing opportunities and challenges for those who take on the carer’s role. 

Ref. [24], in their literature review, claim that the cessation-of-caregiving trajectory [13] is an integral part of the caregiving ‘career’ that all former carers experience [25]. When caregiving careers end and the cessation-of-care period begins, bereavement and an adjustment to a new situation may follow, with various implications for personal welbeing (physical and psychological health) and social and economic aspects (income, assets, employment, jobs) [26]. 

Two other studies identify three phases that carers go through during the cessation-of-care period. Ref. [13], studying cessation of care among white Britons shows that life after caregiving follows a distinct three-phase trajectory—the ‘cessation of caring void’, ‘closing down the ‘caring time’, and ‘constructing life after cessation of caring’. The majority experienced a ‘void’ in their lives after they stopped giving care, characterized in several ways. The first is manifested in the sense of loss of purpose; feeling ‘lost’, disequilibrium; or feeling lonely and ‘falling apart’. The second phase, ‘closing down’ the caring time, is described as a ‘wind-down period’ because it involves the phasing out of life as a carer. During this transitional time, routines change, and closure activities are undertaken. The third phase is characterized by ‘constructing life after the cessation of caring’, which involves ‘getting life together’ again and ‘coming up to the surface’. As these transitions are made, post-caregiving life is entered into gradually [13].

The second study on former family carers, conducted in Ireland [27], also identifies three themes: loss of the caregiving world, living in a state of loss, and moving on. The transition is accompanied by a combination of emotions and economic and social concerns that impact health and wellbeing. The findings point to the possibility of increased loneliness and the extension of the carers’ social, emotional, and financial burden on their cessation-of-caring worlds. This final phase of caregiving is a distinct but important period that has not been well studied. Most studies on caregiving, in fact, elide this caregiving period, corroborating an earlier assertion that this is the ‘ignored phase of caregiving careers’ [28] even though it is recognized as a very stressful time [29].

Given the highly limited and sometimes contradictory evidence on cessation-of-care outcomes [27], it is essential to build a sound conceptual framework that can capture the complexities of these outcomes for caregivers. 

## 2. Conceptual Framework

Family relationships may influence the reactions to, and the process of an adjustment in the cessation-of-care period by providing effective, caring, and positive support. Intergenerational relationships generally enhance individuals’ psychological wellbeing throughout their lives [30,31]. Thus, we based our study on the Intergenerational solidarity and conflict models [1,32]. By allowing cessation of care to be studied in the context of social, economic, physical, and emotional outcomes of middle-aged and older adults who had cared for older family members, this conceptual framework elicits additional insights into this stage in life as these caregivers experience it. 

The paradigm of intergenerational family solidarity represents an effort to conceptualize family relations in adulthood and to develop a theory about parent–child dyads in caregiving relations and their aftermath [33], yielding a taxonomy for the description of sentiments, behaviors, and attitudes in family relationships [34,35]. The paradigm was modified in subsequent scholarship in order to accommodate conflict so that the possible negative effects of excessive solidarity and inconsistencies that affect the way family members perceive one another, consequently impacting their willingness to assist one another, might be considered [36,37,38]. The resulting modified paradigm has guided much of the research on adult intergenerational relationships in recent decades [39,40,41]. This conceptual framework is well-suited to studying cessation of care in a multicultural society such as Israel’s because it presents several relevant components, such as solidarity and conflict, that may be reflected in cessation-of-care situations [42,43,44].

**Figure 1:**
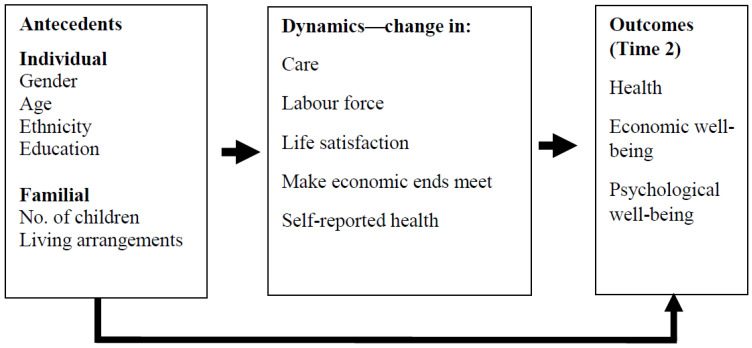
The research model.

The research model that guided the study is presented in Figure 1. With the foregoing kept in mind, the main underlying concept of the research model is the following: the indicators of social welfare, health, and economic wellbeing of older adults who regularly care for a related older adult (in accordance with the conceptual framework presented at length above) are expected to be contingent (both directly and indirectly) upon their background characteristics and are directly contingent on changes that they experience over the years in respect of these indicators, their labor force participation, and whether they change their behavior patterns when they cease to be their relative’s carer.

The results may inform policy on how to support carers and cessation-of-care families and reduce long-term financial problems that have been linked to care and its cessation. Thus, the emphasis on the outcomes of cessation of care for carers addresses an important lacuna in the literature. A second lacuna pertains to the time period: for the most part, studies have focused on short-term adjustments to the cessation of care, whereas the current study uses a longer time frame—around four years. This should yield further insights on lingering effects of care and related variables. We will pay more tribute to the dynamics of care than is typical in care research. The overarching goal of the study is the exploration of cessation-of-care outcomes in three domains of life—psychological, health, and economic—in an aging society.

## 3. Elders and Families in Israel

In Israel, most older adults reside in the community and exhibit a relatively low (4 percent) institutionalization rate. Furthermore, Israel is a family-oriented multicultural society composed of a large part of immigrants from more than one hundred countries. This demographic is more prominent among the aged than among the population at large; thus, uncharacteristically among developed countries, only 28 percent of the older population were born in Israel [45]. Israeli society is composed of two main ethnic groups—Jews (76 percent) and Arabs (24 percent), of whom most are Muslim. The proportion of Arabs in the aged Israeli population is lower than that of Jews, but Arabs have a much higher disability rate. Namely, approximately 30 percent of the older Arab population have disabilities that make them dependent on others for activities of daily living, as against only 14 percent of the older Jewish population [46,47]. 

Despite social transitions from traditional to modern and from collectivistic to individualistic orientations [48,49], older Arabs are more likely than older Jews to live adjacent to or near their adult children. Although filial obligations remain strong, cultural changes are reported, especially with the rising participation of women in the labor market [50]. However, the tendency of women of working age to participate in the labor market remains higher among Jews than among Arabs [51].

The Arab community is in the midst of relatively rapid modernization that may impact filial loyalty and caregiving. Modernization, however, does not necessarily portend the demise of the traditional culture; cultural changes are often accompanied by the retention of traditional values [52,53].

In the past three decades, the Israeli older adult population has been growing at twice the rate of the population at large. The 75+ and, especially, the 80+ group have been expanding with even greater celerity [54]. Given the expectation that this trend will continue, understanding issues related to the family care of disabled elders and its conclusion or cessation is of timely importance. 

In Israel, the gap between demand for and supply of informal and formal care is expected to widen in the coming years [45,55]. Given the increase in longevity, with a life expectancy measured at 81.4 for men and 84.6 for women today [56], many individuals may face multiple transitions into and out of care as aging parents, followed by spouses, and need assistance [57]. These transitions require the renegotiation of family relationships [6,58], sometimes leading to added burden and stress and, in turn, the development of relevant policy and services. 

Israel was the first to enshrine long-term care insurance in the statute [59]. This policy, however, cannot meet all the needs of frail elders and, especially, highly dependent elders. Thus, the family must continue to be involved and provide care. Care recipients and their families do not regard the public services as substitutes for the family and families cannot be replaced because of emotional needs and cultural norms [60]. Thus, families remain the mainstay of elder care in the community. Data from Israel show that 22.5 percent of the adult population serves as informal carers of frail elders at some point in their lives. Among the carers, 38 percent are aged 44–64 [46]. Adult children have been shown to comprise the largest category of membership in elders’ social networks [8]. 

The current study explores the implications of cessation of care by adult family members to frail older adults, comparing two ethnic groups in Israel—Jews and Arabs—with respect to their health, psychological, and economic life. It may generate new insights into the interconnections of health, economic wellbeing, and psychological wellbeing by comparing cessation of care with the care situation, using intergeneration solidarity as the conceptual model. 

## 4. Methods

### 4.1. Data Source and Study Sample 

Data from SHARE—Survey of Health, Aging and Retirement in Europe, based on the US Health and Retirement Study (SHARE project: http://www.share-project.org/ (accessed 15 February 2022)), were subjected to secondary analyses [61]. The purpose of SHARE is to gain a better understanding of the dynamics of the growing population of persons aged 50+ and to serve as a research infrastructure for public policymaking on behalf of the aging population [62]. SHARE is a multi-disciplinary and cross-national survey that yields microdata on health (e.g., self-reported, health conditions, and physical and cognitive functioning), psychological variables (psychological wellbeing, life satisfaction), economic variables (e.g., current work activity, job attributes, sources, and composition of current income), and caregiving (personal care assistance, practical housework, and help with paperwork) for individuals aged 50+, from 2004 onward [39,63,64]. The secondary analyses, based on data from two consecutive waves of SHARE-Israel four years apart (hereinafter—Time 1 and Time 2) are a longitudinal investigation that tracks respondents across these two research periods. The analyses focus on monitoring the provision of informal care among persons aged 50+ who belonged to the population that was interviewed in both waves.

SHARE is harmonized with leading international surveys, such as the US Health and Retirement Study (HRS), the English Longitudinal Study of Aging (ELSA), and the Irish Longitudinal Study on Aging (TILDA). It is also a prime resource for newer surveys of its type in a range of countries, such as Japan (JSTAR), China (CHARLES), India (LASI), and Brazil (ELSI) [61].

The follow-up population comprises 1710 respondents, of whom 75 percent were aged 50–69 and the others were 70+. In their interviews, they were asked, among other things, whether they had provided assistance to an older family member in the preceding twelve months. Two questions were asked: ‘Is there someone living in this household whom you have helped regularly during the last twelve months?’ and ‘Have you personally given any kind of help to a family member from outside the household in the last twelve months?’ The participants were then asked about at least one of the three following types of assistance: (a) personal care, such as dressing, bathing, or showering, feeding, getting out of bed, and using the toilet; (b) practical help around the house, such as home repairs, taking care of the garden, transport, shopping, and housework; (c) assistance with paperwork, such as filling in forms or arranging financial or legal affairs.

Based on these variables, a typology of transitions between states of informal care provision was created for the entire research population, including the following transitions: provided informal care in both waves, provided informal care at Time 1 but not at Time 2 (i.e., stopped giving informal care), did not provide informal care at Time 1 but provided it at Time 2 (i.e., began to provide informal care), and did not provide informal care at either time. In the current study, we analyzed two groups only: those who provided help at Time 1 and either continued to do so at Time 2 or stopped doing so (i.e., entered the cessation-of-care phase).

Importantly, the SHARE data does not allow us to pinpoint the exact reasons for cessation of care; we can use them only to track changes in carers’ characteristics over time and estimate whether the change in caring status has significant effects on their physical, psychological, and economic outcomes. SHARE provides no information on what happened to recipients of care after cessation of care—did they die? Did responsibility for their care shift to another family member, or were they placed in institutional care? The absence of this information does not diminish the importance of the research model and the findings presented below because the current study focuses on events related to family members who provide the care and not on those who receive it. 

### 4.2. Research Variables

#### 4.2.1. Dependent Variables

The dependent variables were measured at Time 2 in order to take advantage of the longitudinal structure of the database to see if changes in the explanatory variables impact the dependent variables. The variables included:

(a) State of health—self-rated health (1 = very bad, to 5 = excellent);
(b) An economic variable—subjective evaluation of household economic wellbeing: making ends meet (1 = with great difficulty to 4 = easily);
(c) Psychological wellbeing—life satisfaction (0 = not at all satisfied to 10 = very satisfied).

#### 4.2.2. Explanatory Variables

(a) ‘Stop giving care’ (cessation of care): 0 = helped an older family member at both Time 1 and Time 2; 1 = helped an older family member at Time 1 and stopped care at Time 2.
(b) Socio-demographic attributes: age, gender (0 = female, 1 = male), living arrangements (0 = living alone, 1 = living with others), children (0 = none, 1 = having), years of education, and ethnicity (0 = Jewish, 1 = Arab). Data regarding all socio-demographic explanatory variables were harvested from Time 1 only.
(c) Interaction variables between the different socio-demographic attributes and cessation-of-care opportunities were also included. This allowed us to identify the combined effect of the two variables on each of the research variables in the model, yielding a subjective picture of the care process before it began, when it was in place, and after it was over.

According to the literature, the changes that a person experiences over the years in regard to the variables studied are indicative of the intensity of the inner change that the researched person undergoes during the investigation period—and it is this intensity indicator that should explain the individual’s pattern of behavior in the later portion of his or her life [62]. This method, which uses change variables to estimate behavior patterns longitudinally, is accepted and practiced in research that tracks individuals across periods when the aim is to determine whether the intensity of a change in a given variable contributes above and beyond all other variables studied in relation to the later variable (see, for example, ref. [65]). Based on that theoretical background, we included the following explanatory variables:

(d) Changes in carers’ labor force participation between the caregiving period and the cessation-of-care period (−1 = left labor force, 0 = no change, 1 = enter labor force).
(e) Changes in the aforementioned dependent variables (self-rated health, life satisfaction, making ends meet) between the caregiving period and the cessation-of-care period, a higher score signifying a positive change.

## 5. Results

Most informal care was provided to mothers/mothers-in-law (73.9 percent), followed by care for fathers/fathers-in-law (17.5 percent). A much smaller percentage of respondents helped spouses/partners or siblings (8.6 percent). All three types of care—personal care, practical household help, and help with paperwork—were provided mostly to mothers/mothers-in-law (Table 1).

Table 2 reports our bivariate analysis of the socio-demographic attributes and subjective evaluation of health, life satisfaction, and financial situation for each of the two groups of carers—those who stopped giving care and those who continued giving care between Time 1 and Time 2 of the longitudinal data used. The results show that the dependent variables—self-rated health and life satisfaction—were significantly higher within the group that continued care. Two explanatory variables revealed significant differences (1) age—those ceasing care are older than those who continue to provide it, and (2) labor force participation—those who cease providing care had a much lower rate of labor force participation (at Time 1) than did those who continue providing it. The latter difference may trace to disparities in age and in the assessment of health among the members of these two groups. In all the other variables (e.g., household economic wellbeing, gender, living arrangements, children, education, and ethnicity, changes in the aforementioned dependent variables between the care period and the cessation-of-care period), there was no significant difference between the groups. 

To examine the impact of ‘stop giving care’ (cessation of care) on the three aforementioned domains of life—self-rated health, economic situation (‘make ends meet’), and life satisfaction—among middle-aged and older adults 50+ who cared for an elderly family member at Time 1, we conducted stepwise regressions using the ordered logit model (OLM) and the linear regression model (OLS), respectively. Stepwise regression is a step-by-step iterative construction of a regression model that selects independent variables for use in the final model by adding or removing potential explanatory variables in succession and testing for statistical significance after each iteration [66]. In each step, a variable is considered for addition to or subtraction from the set of explanatory variables based on some pre-specified criterion [67].

We use an ordered logit regression analysis to produce predicted odds ratios (ORs) for each factor that yield information about a relative increase in the odds of self-rated health assessment or making ends meet being higher among middle-aged and older adults 50+ who cared for an elderly family member at Time 1, adjusted for all other variables in the model. Significant odds ratios that are greater than 1 are interpreted as increasing the probability; those less than 1 are seen as decreasing the probability. We estimated the model using a hierarchical approach, first including socio-demographic background characteristics and then adding changes, followed by the ‘stop giving care’ (cessation of care) factor and, finally, interaction variables. Table 3, Table 4 and Table 5 present the results.

Table 3 reports the analysis of the impact of several variables on self-rated health assessment at Time 2 among middle age and older adults 50+ who cared for an elderly family member at Time 1. In the first step, background attributes were entered, and age (younger), gender (male), and education (higher) were found to have a positive impact on the probability of having a positive self-rated health assessment. In the second model, changes in labor force participation, life satisfaction, and economic situation were entered, and these variables were also shown to impact the self-rated health assessment positively. The third model introduced the variable of ‘stop giving care’ (cessation of care), which impacted positively above and beyond the other variables. By entering the ‘stop giving care’ variable, however, we found that gender is irrelevant and that ethnicity—being Arab—impacts health assessment negatively. The last-mentioned outcome may trace to structural disparities in access to and supply of healthcare services in Israel between localities inhabited by Jews and those that have an Arab majority, to the advantage of the former [68]. The last model augments all the preceding models by adding interaction variables between socio-demographic attributes and cessation of care. It emphasizes that the probability of a positive assessment of health among people who stopped giving care declines with age but increases with their having children (in comparison with all other respondents).

Table 4 presents the analysis of the impact of several variables on the economic situation of ‘make ends meet’ at Time 2 among middle age and older adults 50+ who cared for an elderly family member at Time 1. In the first step, when background attributes were entered, gender (male) and education (higher) were found to have a positive impact on the probability of making ends meet easily whereas being Arab or having children had a negative impact. In the second model, changes in life satisfaction were the only change variable that impacted the economic situation positively. The third model introduced the variable of ‘stop giving care’ (cessation of care), which impacted positively above and beyond the other variables. The last model added, beyond all preceding models, interactions between the socio-demographic variables and cessation of care. It emphasizes that the probability of easily making ends meet among people who stopped providing care declines with age and rises with their level of education (in comparison with all other respondents).

Table 5 shows the analyses of the impact of several variables on subjective evaluation of life satisfaction at Time 2 among middle-aged and older adults 50+ who cared for an elderly family member at Time 1. In the first step, when background attributes were entered, being Arab and having a (higher) education were found to have a positive impact whereas age (older) had a negative impact. In the second model, changes in an economic situation and changes in self-rated health impacted the economic situation positively. The third model introduced the variable of ‘stop giving care’ (cessation of care), which impacted life satisfaction negatively, above and beyond the other variables. The last model added, beyond all preceding models, interactions between socio-demographic variables and cessation of care. It emphasizes that satisfaction with life among people who stop giving care correlates positively with their having children and with their level of education and correlates negatively with their age (in comparison with all other respondents).

**Figure 2:**
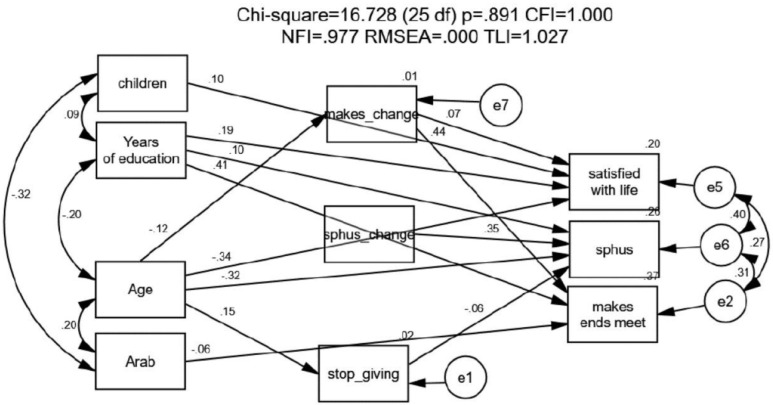
Structural equation model (SEM) analysis of the effect of socioeconomic, changes, and stop giving on life satisfaction, subjective health situation, and the ability to make ends meet. The arrows represent significant associations only.

After estimating characteristics that explain self-rated health, the ability to make ends meet, and life satisfaction as a function of a range of demographic indicators, changes in the other explained variables, and the predisposition to stop giving care, we now turn to the last part of our empirical model. In this stage, we attempt to determine: (a) whether the three explained variables mentioned above are correlated; (b) whether a different pattern of explanation pertains to each variable, and (c) whether other variables mediate between the exogenous variables and the explained ones. Pearson correlations were used to test for correlations among the study variables. The AMOS structure equation modeling (SEM) program, version 25, was run to create a path analysis using the maximum-likelihood method [69,70]. SEM was used to explore paths of the relation between the socio-demographic attributes, the interactions, the changes in variables, the ‘stop giving care’ (cessation of care) explanatory variable, and the outcome variables. The values shown are β values that relate to correlations between variables that were found to be significant (Figure 2).

First, we found (by means of the Pearson-r) a clear correlation among self-rated health, the ability to make ends meet, and life satisfaction. Furthermore, having children and having more years of education amplify life satisfaction, whereas an increase in age reduces it. A positive change in an individual’s ability to make ends meet enhances life satisfaction by the very fact of its moderating between the person’s age and his or her life satisfaction. Self-rated health is positively dependent on years of education and negatively dependent on age. ‘Stop giving care’ moderates the relation between individuals’ age and their self-rated health, which is better among those who do not stop giving care. Finally, an increase in years of education enhances individuals’ ability to make ends meet, whereas being Arab has the opposite effect.

## 6. Discussion

The study investigated the cessation of informal care for older family members among the middle-aged and older adult (50+) population in Israel by using secondary longitudinal analyses of SHARE-Israel data. It sought to understand the set of factors that explain three outcome aspects: health, economic situation, and psychological wellbeing. Physical health was judged through the prism of self-rated health, the economic situation was measured by the evaluation of ‘making ends meet’, and psychological wellbeing was gauged by the evaluation of life satisfaction.

The study was based on a panel series among persons aged 50+ in Israel over a four-year research period. A salient advantage of this type of monitoring is the possibility of examining a broad range of issues on the basis of participants’ longitudinal personal responses—from demographic and economic indicators and estimations of psychological wellbeing to patterns of the subjectively rated state of health.

We developed a stepwise model to analyze antecedents and outcomes of the complicated dynamics of the care process among middle-aged children, mainly for parents, and its cessation. The model covered four types of elderly kin (mothers, fathers, spouses, and older siblings) and three areas of care (personal care, practical household help, and help with paperwork). Antecedents included individual socio-demographic attributes, such as age, gender, ethnicity, education, and familial characteristics: number of children and type of living arrangements. 

Most research produces a snapshot in time or captures cessation-of-caring experiences over a relatively short period, whereas we used two waves of SHARE data that were collected at two junctures four years apart. The dynamic aspects of the model related to care transitions reflected changes in the carers’ health, economic, and life satisfaction after they ceased to provide care. The significant outcomes of the study indicate the importance of these components for enhancing our understanding of the care process and the implications of cessation of care for important areas of carers’ lives. 

Informal caregiving by a family member is an intensive experience that may be conceptualized as a career, as caregivers may repeatedly enter and exit this role and alter the amount of care they provide [71]. Intergenerational exchange is an important social issue [72] because families in modern society are still the main source of care and support for older people [73].

Our study was based on the intergenerational solidarity and conflict models [1,32] and the intergenerational family solidarity paradigm [33]. The solidarity model is a taxonomy for describing sentiments, behaviors, and attitudes in family relationships [34,35]. On this basis, the innovative contribution of this study is its focus on the dynamic process in intergenerational care relations, in which a transition is made from being an active caregiver for an old family member to the cessation of care and its impacts. Family intergenerational relations of solidarity and conflict may influence the responses and the adjustment process that unfolds in the cessation-of-care period.

This final phase of care is a distinct but important period that has not been well studied. Few longitudinal studies include the cessation-of-care period, supporting an earlier assertion that this is the ‘ignored phase of care careers’ despite its recognition as a highly stressful event [29,74]. It is imperative to study this topic ‘in a dynamic design and in different countries … which will increase insight in the developments of informal care provision’ [75]. 

Changes in caregiving status and intensity influence carers’ physical, economic, and emotional wellbeing. Few studies, however, have evaluated the simultaneous impact of these changes on carers’ lives. For example, [76] studied the impacts of informal caregiving on caregivers in three areas—employment, health, and family—but did not relate to the cessation-of-care period. In their conclusions, they point to methodological limitations of caregiving studies, for example, non-representative or small samples and limited use of control variables and cross-sectional analyses [76]. In this study, we tried to overcome some of these shortcomings by using representative samples, a wide variety of control variables, and a longitudinal design. However, secondary analysis of existing data, like SHARE data, imposes some restrictions, such as limiting the investigation of life satisfaction to one question only. 

The goal of this study was to explore the implications for middle-aged and older adult family members of stopping care of disabled elders (entering the cessation-of-care phase). 

Two main conclusions can be drawn. First, when the group that continued to provide care at Time 2 was compared with the other, which stopped giving care, the group that continued care showed significantly higher subjectively rated health and life satisfaction; it was also found to be younger and its participation in the labor force was higher. 

Second, ceasing to provide care has significant implications for all three areas studied; subjectively rated health and financial capabilities improved. An extensive literature review on caregiving impact offers strong evidence that carers are more likely to work fewer hours as they adjust to the caregiving situation [76], lowering their wages and pension contributions and, in turn, compromising their post-retirement income [27,77]. In recent findings, [78] confirm that the combination of intensive caregiving and labor force participation increases the risk of negative physical and mental health effects. Additionally, providing informal care to an older adult has adverse impacts on carers’ state of health.

Thus, it is reasonable to infer that after the cessation-of-care period, carers would be involved in what [13] showed as ‘constructing life cessation of caring’, in which paid employment plays a significant part and allows for higher wages. Additionally, as providing informal care to an older adult has adverse impacts on carers’ state of health, there is one way to assume that their health situation will improve after the care phase has ended. However, there is no universal negative impact of caring on health. Where caring impacts health, it is likely to do so through stress. This means that life satisfaction would be more sensitive to this effect in the short to medium term than would self-rated health. It also means that we should expect to see an improvement in life satisfaction following cessation. Cessation of care may involve negative emotions and bereavement, which have the capacity to impact negatively on health. Therefore, theory and literature do not point unequivocally to better health among those who have recently ceased providing care.

The carers in our study, however, reported a decline in life satisfaction after they stopped giving care. This squares with data from [13] qualitative study, which identified three cessation-of-care phases, including a cessation-of-caring void [79] that arouses emotions of ‘guilt, loneliness and loss of esteem and purpose’. This may suggest possible reasons for the decline in carers’ life satisfaction: the death of the care recipient or deterioration in health that forces carers to hire a formal worker and/or place the person being cared for in an institution, resulting in loss of the role of carer. Another dimension that [13] found among her interviewees was a feeling of loneliness due to the loss of social networks of health and welfare professionals. However, [74] in their comparison of caregivers and those who stopped giving care in terms of stress levels, found that regardless of intensity level, those who stopped care reported the same amount of stress as those who continued to give care. Thus, it would be of interest to ask whether such a decline in life satisfaction and its implications are short- or long-term and to detect the determinants of such a process.

‘Variation in informal care among countries’, ref. [76] state, ‘can be accounted for at least to some extent by differences in the availability of public support which determines the opportunity costs of becoming a caregiver’. For example, many welfare states nowadays try to support family carers by providing cash benefits, as is done in a third of OECD countries; in-kind benefits, as in Israel [59]; or a combination of the two, as in Germany. Our results also demonstrate the need to support those who exit the role of carer for frail family elders.

To fully understand cessation-of-care outcomes, as explained above, one must consider the developmental timing of the transitions, the parallel trajectories in other areas of the carer’s life (health, family, career), and factors that moderate or mediate adjustment once the care has ended. One limitation is noteworthy in this context. The SHARE data does not allow us to pinpoint the exact reasons for cessation of care; they let us only learn about changes in carers’ characteristics over time and estimate whether the change in caring position has significant effects on their physical, psychological, and economic outcomes. It is also noteworthy that SHARE yields no information about when the relative began to provide care and the intensity of the care given. Consequently, we are unable to discuss the question of the ‘price’ of caregiving and its cessation as a function of these two indicators.

## 7. Conclusions

This study, based on the intergenerational solidarity and conflict models [36,37,38] as well as the paradigm of intergenerational family solidarity [33,34,35], breaks new ground by presenting a dynamic perspective on understanding the implications of ceasing to provide care for the lives of carers of elderly kin, even though the share of the elder population that receives assistance and care from a family member has been steadily rising over the years as the proportion of elderly in the country’s population climbs. Welfare states, too, are finding it hard to cope with the tasks flowing from the need to care for the elderly population, resulting in a burden on family members—particularly those of the ‘sandwich generation’. This burden evolves into a role identity among some caregiving families, one that family members may ‘enter’ and ‘exit’ several times in the course of their adult lives.

Although cessation of care was found to have a positive impact in two life domains—state of health and economic situation—a negative effect was found in one respect, life satisfaction. This situation points to the need to provide carers with support and targeted intervention strategies. 

In view of the overall findings of this study, the next stage of our research should be devoted to a thorough understanding of the care process and the factors that prompt informal carers to stop caring for elderly kin. This aim cannot be accomplished using the SHARE data; it will be achieved through the use of qualitative methods in future research. 

The caregiving experience, coping with the role and its demands along with other tasks in caregiving family members’ lives, and the implications of cessation of care for carers’ health, economic wellbeing, and life satisfaction are aspects that we may address seriously with a depth and specificity that research has not yet offered, yielding insights on the determinants of cessation of informal care. This stage of research is likely to be carried out in a further empirical study. Its implementation will produce a profound and thorough complement to the scientific picture reflected in the current study, yielding a lucid and comprehensible understanding of references to the question of stopping infomal care of elder family members.

## Figures and Tables

**Table 1:** Characteristics of informal care provided (percent).

	Distribution of Informal Care Recipients	Type of Informal Care Provided		
		Personal Care	Practical Household Help	Help with Paperwork
Mother/mother-in-law	73.9	60.0	74.8	76.0
Father/father in-law	17.5	23.7	16.9	15.7
Spouse/partner/siblings	8.6	16.3	8.3	8.3
**Total**	**100.0**	**100.0**	**100.0**	**100.0**

**Table 2:** Comparison of two carer groups—those who continued providing care, and those who ceased (means and percentages) ^1,2^.

		Time 1—Giving; Time 2—Not Giving	Time 1—Giving; Time 2—Giving	F/χ^2^
**Dependent variables**				
Self-reported health (mean)		2.57	3.11	10.12 ***
		−1.13	−1.04	
Household makes ends meet (mean)		2.62	2.83	1.37
		−1.04	−0.99	
Life satisfaction (mean)		6.79	7.85	6.32 ***
		−3.25	−1.94	
**Explanatory variables**				
Age (mean)		67.83	64.15	10.91 ***
		−10.10	−7.52	
Gender (pct.)	Female	58.21	56.24	0.07
	Male	41.79	43.76	
Living Status (pct.)	Living alone	24.26	25.39	0.16
	Living with someone	75.74	74.61	
Children (pct.)	No	4.37	5.83	0.13
	Yes	95.63	94.17	
Ethnicity	Jews	79.11	81.14	0.00
	Non-Jews	20.89	18.86	
Education (mean)		12.76	13.77	2.11
		−4.23	−4.33	
Self-reported health—change (mean)		−0.35	−0.34	0.01
		−0.94	−0.91	
Life Satisfaction—change (mean)		−0.29	−0.09	1.00
		−1.69	−1.47	
Household makes ends meet—change (mean)		0.21	0.28	0.08
		−0.97	−0.98	
Labor force (pct.)	Non-participants	62.44	50.21	4.49 **
	Participants	37.56	49.79	
Labor force—change (pct.)	Left labor force	11.28	13.23	0.04
	No change	86.19	83.92	
	Enter labor force	2.53	2.84	

Significance levels: ** *p* < 0.05; *** *p* < 0.01. Note: ^1^ Figures in parentheses are standard deviations. Values are based on weights of Time 1. ^2^ The table presents only the significant variables in the bivariate analysis.

**Table 3:** Ordered logistic regression analysis with self-rated health as a dependent variable (odds ratio).

	Model 1		Model 2		Model 3		Model 4	
	Odds Ratio (Std. Error)	95% CI	Odds Ratio (Std. Error)	95% CI	Odds Ratio (Std. Error)	95% CI	Odds Ratio (Std. Error)	95% CI
Age	0.961 *** (0.00)	[0.954, 0.968]	0.962 *** (0.00)	[0.955, 0.969]	0.963 *** (0.00)	[0.956, 0.970]	0.967 *** (0.00)	[0.959, 0.975]
Male	1.131 ** (0.07)	[1.007, 1.271]						
Arab					0.816 *** (0.06)	[0.701, 0.949]	0.828 * (0.06)	[0.711, 0.964]
Education	1.053 *** (0.01)	[1.037, 1.068]	1.046 *** (0.01)	[1.031, 1.062]	1.028 *** (0.01)	[1.012, 1.043]	1.032 *** (0.01)	[1.016, 1.048]
In labor force—change			1.516 * (0.32)	[0.998, 2.303]	1.522 * (0.33)	[0.998, 2.322]	1.622 * (0.35)	[1.063, 2.477]
Life satisfaction—change			1.236 *** (0.03)	[1.173, 1.303]	1.222 *** (0.03)	[1.158, 1.290]	1.212 *** (0.03)	[1.148, 1.279]
Make ends meet—change			2.175 *** (0.13)	[1.933, 2.447]	2.114 *** (0.13)	[1.872, 2.386]	2.071 *** (0.13)	[1.834, 2.338]
Stop giving care					1.234 *** (0.02)	[1.188, 1.282]	1.228 *** (0.02)	[1.182, 1.275]
Age*Stop giving care							0.975 *** (0.01)	[0.961, 0.989]
Children*Stop giving care							3.109 * (1.53)	[1.185, 8.158]
Log-likelihood	−5419.8506		−5291.3720		−4975.1555		−4958.6279	
N	1471		1462		1282		1282	

Source: Survey of Health, Aging and Retirement in Europe (SHARE)-Israel. Significance levels: * *p* < 0.1, ** *p* < 0.05, *** *p* < 0.01. Standard errors are in parentheses. The models were estimated using the stepwise method. Another variable included but not significant is living with children.

**Table 4:** Ordered logistic regression analysis with ‘make ends meet’ as dependent variable (odds ratio).

	Model1		Model 2		Model 3		Model 4	
	Odds Ratio (Std. Error)	95% CI	Odds Ratio (Std. Error)	Odds Ratio (Std. Error)	Odds Ratio (Std. Error)	95% CI	Odds Ratio (Std. Error)	95% CI
Male	1.166 ** (0.07)	[1.035, 1.314]	1.133 ** (0.07)	[1.005, 1.278]	1.127 * (0.07)	[0.995, 1.277]	1.125 (0.07)	[0.991, 1.277]
Arab	0.506 *** (0.04)	[0.435, 0.588]	0.486 *** (0.04)	[0.418, 0.566]	0.672 *** (0.06)	[0.571, 0.790]	0.659 *** (0.06)	[0.559, 0.777]
Children	0.684 *** (0.07)	[0.553, 0.847]	0.716 *** (0.08)	[0.577, 0.888]	0.726 *** (0.08)	[0.581, 0.907]	0.717 ** (0.08)	[0.574, 0.896]
Education	1.135 *** (0.01)	[1.118, 1.152]	1.146 *** (0.01)	[1.129, 1.163]	1.117 *** (0.01)	[1.100, 1.135]	1.109 *** (0.01)	[1.091, 1.128]
Life satisfaction—change			1.514 *** (0.07)	[1.382, 1.658]	1.407 *** (0.07)	[1.280, 1.547]	1.484 *** (0.12)	[1.265, 1.741]
Stop giving care					1.456 *** (0.03)	[1.399, 1.516]	1.459 *** (0.03)	[1.401, 1.519]
Age*Stop giving care							0.982 *** (0.00)	[0.972, 0.992]
Education*Stop giving care							1.110 *** (0.03)	[1.061, 1.161]
Log-likelihood	−4670.9813		−4611.5884		−4196.3078		−4185.505	
N	1475		1463		1283		1283	

Source: Survey of Health, Aging and Retirement in Europe (SHARE)-Israel. Significance levels: * *p* < 0.1, ** *p* < 0.05, *** *p* < 0.01. Standard errors are in parentheses. The models were estimated using the stepwise method. Other variables included but not significant are age, living with, self-rated health—change, and in the labor force—change.

**Table 5:** Linear regression analysis with life satisfaction as a dependent variable.

	Model 1		Model 2		Model 3		Model 4	
	Coefficient (Std. Error)	95% CI	Coefficient (Std. Error)	95% CI	Coefficient (Std. Error)	95% CI	Coefficient (Std. Error)	95% CI
Age	−0.011 *** (0.00)	[−0.018, −0.004]	−0.009 *** (0.00)	[−0.016, −0.002]	−0.007 * (0.00)	[−0.014, 0.000]		
Education	0.061 *** (0.01)	[0.047, 0.074]	0.061 *** (0.01)	[0.045, 0.074]	0.064 *** (0.01)	[0.050, 0.077]	0.054 *** (0.01)	[0.040, 0.068]
Arab	0.369 *** (0.07)	[0.231, 0.506]	0.364 *** (0.07)	[0.227, 0.501]	0.384 *** (0.07)	[0.247, 0.520]	0.360 *** (0.07)	[0.226, 0.494]
Make ends meet—change			0.085 ** (0.04)	[0.016, 0.154]	0.097 *** (0.04)	[0.029, 0.166]	0.076 * (0.03)	[0.009, 0.144]
Self-rated health—change			0.099 *** (0.03)	[0.040, 0.158]	0.092 *** (0.03)	[0.033, 0.150]	0.094 ** (0.03)	[0.036, 0.152]
Stop giving care					−0.485 *** (0.11)	[−0.691, −0.278]	−0.325 * (0.96)	[−0.545, −0.194]
Age*Stop giving care							−0.083 *** (0.01)	[−0.103, −0.063]
Children*Stop giving care							1.987 *** (0.54)	[0.933, 3.040]
Education*Stop giving care							0.083 *** (0.02)	[0.036, 0.129]
Constant	7.429 *** (0.27)	[6.906, 7.952]	7.340*** (0.27)	[6.818, 7.862]	7.178 *** (0.27)	[6.653, 7.703]	6.884 *** (0.10)	[6.683, 7.084]
R^2^-Adjusted	0.0329		0.0360		0.0411		0.0677	
N	1474		1461		1461		1461	

Source: Survey of Health, Aging and Retirement in Europe (SHARE)-Israel. Significance levels: * *p* < 0.1, ** *p* < 0.05, *** *p* < 0.01. Standard errors are in parentheses. The models were estimated using the stepwise method. Other variables included but not significant are male, living with children, and in the labor force—change.

## Data Availability

Data was obtained from SHARE and are available at http://www.share-project.org/home0.html (accessed on 15 February 2022) with the permission of SHARE.
